# 2849. Doxycycline for the Treatment of Urinary Tract Infections

**DOI:** 10.1093/ofid/ofad500.2459

**Published:** 2023-11-27

**Authors:** Tina Zheng, Monica Mehta, Allison Stilwell, Nicholas Demenagas

**Affiliations:** Maimonides Medical Center , Queens, New York; NewYork-Presbyterian Hospital, Columbia University Irving Medical Center, New York, New York; NewYork-Presbyterian Brooklyn Methodist Hospital, Brooklyn, New York; NYP/Columbia University Irving Medical Center, New York, New York

## Abstract

**Background:**

The 2010 IDSA uncomplicated cystitis and pyelonephritis guideline does not recommend doxycycline as an oral option for the treatment of urinary tract infections (UTI). Doxycycline is reported to have 35-60% urinary excretion. Small prospective cohort studies from the 1960-1980s suggest favorable clinical and microbiological outcomes. This multicenter, retrospective study aimed to describe the effectiveness and safety of doxycycline to treat UTIs.

**Methods:**

Patients aged ≥ 18 years who received at least one dose of doxycycline for the treatment of UTI from September 1, 2020 to August 31, 2022 were included. Patients with a positive chlamydia/gonorrhea result, suspected concomitant sexually transmitted infections, asymptomatic bacteriuria, or with urine culture isolates resistant to tetracyclines were excluded. Treatment success was defined as resolution of urinary symptoms on day 3 and the last day of treatment for patients who received their entire doxycycline course while inpatient. Secondary outcomes included clinical recurrence, microbiological success and recurrence, and adverse drug events. Patients who had a partial inpatient doxycycline course met treatment success criteria if they did not re-present for medical care with UTI symptoms within 7 days post-treatment completion.

**Results:**

Seventeen patients were evaluated. Patients were older with a median age of 65 years, and 47% reported antibiotic allergies. Most patients had cystitis (65%), though four patients had pyelonephritis (24%). The most common organism isolated was Klebsiella pneumoniae (24%). Resistance to third-generation cephalosporins, carbapenems, and levofloxacin was 79%, 14%, and 53%, respectively. The median duration of doxycycline treatment was 8 days. Treatment success for the complete inpatient treatment group and the partial inpatient treatment group was 75% (6/8) and 89% (8/9), respectively. Only one patient (6%) experienced syncope attributed to doxycycline.

**Conclusion:**

Despite the limited urinary penetration of doxycycline, this study suggests that it may be an alternative option for treatment of UTIs, particularly in patients with a lack of oral options due to allergies or resistant organisms.

**Disclosures:**

**All Authors**: No reported disclosures

